# Automatic Fall Monitoring: A Review

**DOI:** 10.3390/s140712900

**Published:** 2014-07-18

**Authors:** Natthapon Pannurat, Surapa Thiemjarus, Ekawit Nantajeewarawat

**Affiliations:** 1 Sirindhorn International Institute of Technology, Thammasat University, Pathumthani 12121, Thailand; E-Mails: p_natthapon@yahoo.com (N.P.); ekawit@siit.tu.ac.th (E.N.); 2 National Electronics and Computer Technology Center, Pathumthani 12120, Thailand

**Keywords:** fall monitoring, fall detection, fall prevention, wireless sensors, wearable sensors

## Abstract

Falls and fall-related injuries are major incidents, especially for elderly people, which often mark the onset of major deterioration of health. More than one-third of home-dwelling people aged 65 or above and two-thirds of those in residential care fall once or more each year. Reliable fall detection, as well as prevention, is an important research topic for monitoring elderly living alone in residential or hospital units. The aim of this study is to review the existing fall detection systems and some of the key research challenges faced by the research community in this field. We categorize the existing platforms into two groups: wearable and ambient devices; the classification methods are divided into rule-based and machine learning techniques. The relative merit and potential drawbacks are discussed, and we also outline some of the outstanding research challenges that emerging new platforms need to address.

## 1. Introduction

Falls represent one of the leading causes of deaths and injuries in the elderly population. According to Lord *et al.* [1], more than one-third of home-dwelling people aged 65 or above and two-thirds of those in residential care fall one or more times each year. More than two-thirds of people who have experienced a fall are prone to falling again [2]. Vellas *et al.* [3] reported that 219 out of 487 elderly subjects had experienced a fall during a two-year study period and one-third of which developed a fear of falling after the incident. The psychological consequences often lead to decreased mobility and independence among elderly population [4]. Falls can occur on level surfaces, mostly in living rooms, bedrooms, kitchens, bathrooms, or hallways [5]. The rate of fall-related injuries is generally higher among women [6] and the medical costs increase rapidly with age [7]. Damages caused by falls include tissue injuries, lacerations, joint dislocations, bone fractures and head trauma. Carroll *et al.* [8] reported that the total direct medical costs of fall injuries among elderly people in the U.S. in 1997 were $6.2 billion. The costs increased to $19 billion in 2000 [7] and $30 billion in 2010 [9]. Fall-related injury is considered one of the 20 most expensive medical conditions among community-dwelling elderly population [7]. Most elderly people are unable to get up by themselves after a fall and it was reported that, even without direct injures, half of those who experienced an extended period of lying on the floor (>1 h) died within six months after the incident [10].

Fall is defined as “an event which results in a person coming to rest inadvertently on the ground or other lower level”. This definition has been used as a baseline in many fall prevention and fall-risk assessment studies [11–14], and covers most types of falls targeted by fall detection research. Variations of fall definitions from different perspectives of seniors, health care providers and research communities can be found in [15]. Thus far, there are several review papers on fall detection and prevention. Noury *et al.* [16,17] reported a short review on fall detection methods and proposed a set of protocols to evaluate fall detection algorithms. In the study, a fall is divided into four phases, *i.e.*, prefall, critical (impact), postfall and recovery phases, and fall detection algorithms are categorized based on whether they focus on “direct” detection of the critical phase or postfall phase. The critical phase, which consists of a sudden body movement towards the ground, lasts for approximately 300–500 ms.

In 2008, Yu [18] presented a survey on approaches and principles of fall detection. Based on the initial state, fall characteristics are divided into three classes, *i.e.*, fall from sleeping, fall from sitting, and fall from walking or standing. The detection methods are categorized based on device types into wearable, vision, and ambient devices. In 2009, Perry *et al.* [19] provided a brief survey on methods for real-time fall detection, categorizing them into methods that measure only acceleration, methods that use combination of sensors and methods that do not measure acceleration. It was also observed that every fall had a negative peak in the acceleration data; moreover, the acceleration change from positive to negative values and the speed of this change were important for fall detection.

In 2010, Hijaz *et al.* [20] presented another short survey on fall detection and daily activity monitoring, categorizing them into vision-based, ambient-sensor-based, and kinematic-sensor-based approaches. The relative weaknesses and strengths of each approach were discussed. In 2012, Mubashir *et al.* [21] presented another survey following the similar categorization, but with more detailed subcategories. Bai *et al.* [22] described the characteristics of a fall based on three parameters: weightlessness, impact, and overturning of the body. Weightlessness usually occurs at the beginning of a fall. During this period, the acceleration values along three axes are close to 0 g. This is followed by an impact which generates the peak signal, the value of which is usually greater than 1.8 g. Overturning of the body after a fall can be determined by the difference in acceleration signals along the three axes before the weightlessness stage and after the fall.

Recently, El-Bendary *et al.* [23] described causes and consequences of elderly falls, presented the contributions and challenges of existing fall detection techniques, and provided a review of commercial products for fall detection and prevention. In this study, existing commercial products are categorized as wearable and hand-held solutions, movement sensing solutions, and anti-wandering solutions. None of the aforementioned survey provides details on implementation and evaluation that enable quantitative comparison across different systems. Hegde *et al.* [24] published a fall detection review classifying the technologies into four approaches, adding combined wearable and ambient sensors as another separate approach. Only eight wearable-sensor-based fall detection studies were compared in terms of sensor types, sensor placements and performance measures. Igual *et al.* [25] presented a survey of selected 66 out of 327 research publications on fall detection conducted during 2005 to 2012, categorizing them as context-aware-, wearable-accelerometer- and smartphone-based systems.

Although several studies have tried to establish a common evaluation benchmark, only certain aspects of the systems have been addressed. The purpose of this paper is to provide a comprehensive overview of fall monitoring techniques, related commercial products, existing research problems as well as future trends. We will provide an in-depth review of wearable-sensor-based fall detection experiments. The rest of the paper is structured as follows: Section 2 provides a generic overview of fall detection systems and presents the timeline of key features in fall detection research with representative studies. Section 3 summarizes and compares important aspects of wearable fall detection systems. Section 4 presents a summary of commercial products related to fall detection/monitoring. Section 5 describes the future research trends. Section 6 concludes this paper.

## 2. Fall Detection Systems

**Figure 1.**
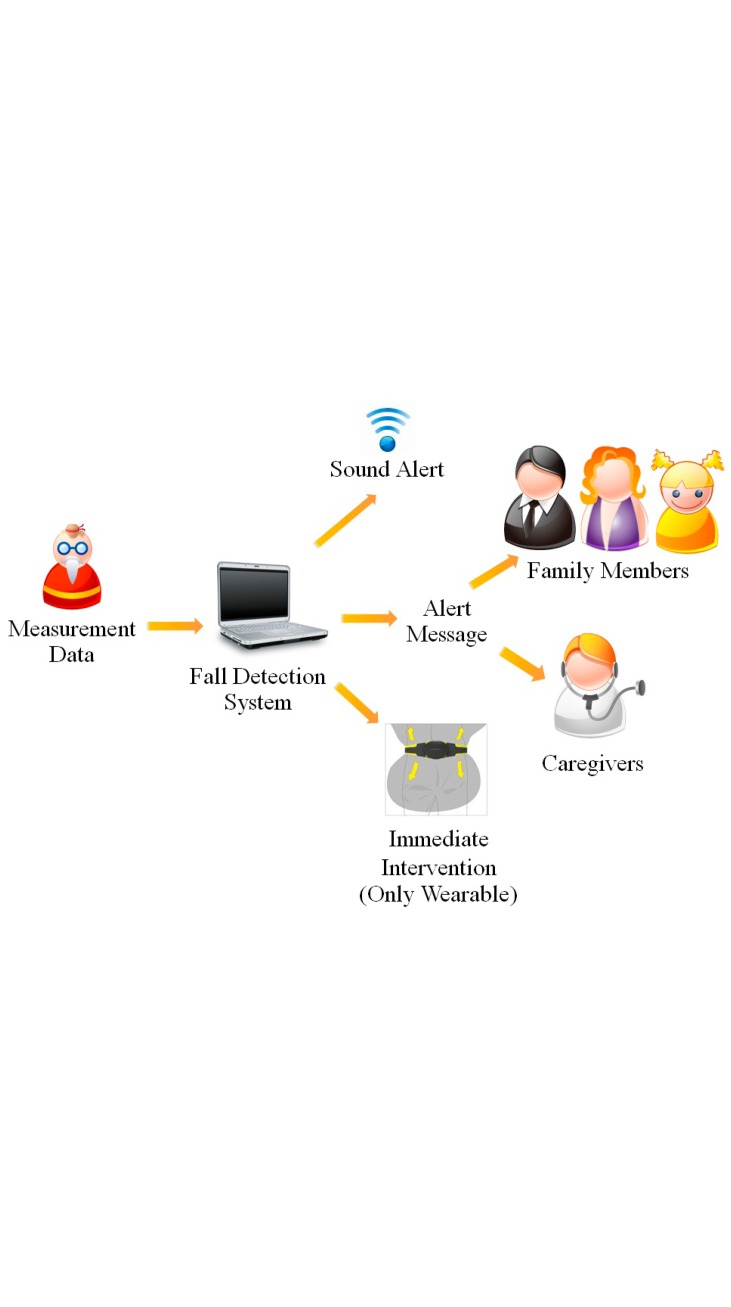
A system overview of a typical fall detection system.

Figure 1 depicts a typical fall detection system. The flow of real-time fall detection system starts with the detection device(s) sending motion data to a processing unit. When the algorithm captures a fall, alarm/action will be triggered. This can be in forms of alert sound (to attract attention and help from people in the vicinity), immediate intervention (e.g., inflating an airbag), or sending alarm messages to family members and/or caregivers. The information may also include time of the incident, location, direction, and status (conscious/unconscious) of the faller.

The first fall monitoring system was developed in the early 1970s. It was designed to send out an alert message when a remote transmitter button was pressed by the user [26]. Automatic fall monitoring research, on the other hand, has been conducted since 1990s. Lord and Colvin [27] studied causes and effects of falls in elderly population in an attempt to prevent falls and proposed the use of an accelerometer for fall detection. The first prototype system was developed in 1998 by William *et al.* [28], using a piezoelectric shock sensor to detect the abnormal peak due to falling and a mercury tilt switch to detect the orientation of the wearer after a fall. One of the first fall detection experiments was based on video cameras. Gu [29] conducted an experiment using three Broadcast Television Systems (BTS) and illustrated that horizontal and vertical velocities can be used to distinguish falls from normal activities. In 2002, Prado *et al.* [30] developed a prototype of fall detection system based on two dual-axial accelerometers worn as a patch on the back of the user at the height of the sacrum. Noury [31] developed a smart fall sensor consisting of a piezoelectric accelerometer, a position tilt switch, and a vibration sensor. A simple algorithm was presented, however, the results showed that the algorithm was too sensitive. Degen *et al.* [32] presented a wrist-worn fall detector for elderly population. The device was easy to wear but yielded only 65% sensitivity. Sixsmith *et al.* [33] used an array of low-cost infrared cameras mounted on the wall. An alarm was triggered either by an excessive period of inactivity or detection of a fall. Experiments on 20 fall and 10 non-fall scenarios performed by an actor, however, showed that the system could detect only 30 percent of actual falls. In 2006, Kang *et al.* [34] developed a wrist-worn integrated health monitoring device consisting of a fall detector and modules for measuring single-channel electro-cardiogram (ECG), noninvasive blood pressure (NIBP), pulse oximetry (SpO2), respiration rate, and body surface temperature (BST). Nyan *et al.* [35] conducted fall detection experiments based on a combined use of a high speed camera and three gyroscopes embedded within a garment near the chest, trunk (below the armpit), and waist. The camera was used to study the body configuration during a fall while the angular rate was used for fall detection. Miaou *et al.* [36] conducted fall detection based on an omni-camera and personal information (e.g., ratio between height and width and a body mass index) stored in a database. The system actually performed lying detection and yielded an accuracy of 70% and 81% with and without personal information, respectively. Alwan *et al.* [37] designed a fall detection system based on floor vibration using a piezoelectric sensor. Even though 100% detection rate was reported, the fall events were simulated using anthropomorphic dummies.

In 2007, Srinivasan *et al.* [38] studied automatic fall detection based on a tri-axial accelerometer and Passive Infrared Sensors (PIRs). The wearable tri-axial accelerometer was placed on the subject's waist to capture fall events while the PIRs were mounted on the wall to provide longitudinal motion information. Motionless signals from PIR sensors were used to confirm fall events. A wearable sensor was not only placed on the subject's body, but also placed on an assistive device. Almeida *et al.* [39] presented a walking stick with a gyroscope embedded at its base for detecting fall and measuring walking pace. A fall was detected based on the magnitude of the resultant angular velocity along sideward and forward axes. The pace was derived from the summation of angular velocity between two adjacent peaks divided by the time interval between the two peaks. Warnings were given when a user walks faster than his/her normal speed. Lin *et al.* [40] performed fall detection based on an optical sensor and nine micro mercury switches embedded into a smart coat. The optical sensor placed on the left waist was used to detect a fall, while the micro mercury switches were used to identify the fall characteristics (*i.e.*, forward or backward) and user's activities (*i.e.*, lying, sitting, standing, or forward bending).

In 2008, Doukas and Maglogiannis [41] proposed a combined use of an accelerometer and a microphone placed on the foot. Based on short-time Fourier transform, it was reported that low frequency sound signals were generated with high amplitude during ground impact and could be used to detect fall events. Grassi *et al.* [42] combined the use of a 3D time-of-flight camera, a wearable Micro-Electro-Mechanical Systems (MEMS) accelerometer, and a microphone for fall detection. Three sensors integrated on a custom board were separately processed and evaluated with suitable algorithms. Popescu *et al.* [43] proposed to use an array of acoustic sensors for fall detection. In 2010, Rimminen *et al.* [44] presented the use of near-field imaging floor sensors for fall detection. Fall classification was performed using a two-state Markov chain and pose estimation based on Bayesian filtering. Tzeng *et al.* [45] used a floor pressure sensor for detecting high impact on the floor and an infrared camera to identify subject's actions. Bianchi *et al.* [46] developed their fall detection system based on a barometric pressure sensor and a tri-axial accelerometer placed on the waist. Based on the assumption that the atmospheric pressures between the waist and the ground are different, the experimental results showed that the sensor information can provide useful information towards fall detection. Dai *et al.* [47,48] introduced the use of mobile-phone-based fall detection. In 2011, Gjoreski *et al.* [49] performed fall detection by placing tri-axial accelerometers on the chest, waist, thigh, and ankle. They reported that sensor placement on the chest or waist was suitable for fall detection but a combination of four sensors yielded the best performance. Li *et al.* [50] detected falls by using accelerometers placed on different parts of the body (*i.e.*, chest, waist, wrist, thigh, ankle) combined with information derived from sensors placed on furniture (*i.e.*, bed and chair).

Recently, fall detection based on tri-axial accelerometers embedded in smart phones has become increasingly popular. Fang *et al.* [51] compared the accuracy of fall detection based on a smart phone positioned on the waist, chest and thigh and reported that the chest is the optimal placement. The advantage of using a smart phone for fall detection is that it can also be used to send out warning messages and/or track the location of a faller. Koshmak *et al.* [52] conducted a fall detection experiment on seven novice skiers by asking each of them to carry a smart phone while skiing. Heart rate and oxygen saturation were also measured during the experiment, and unexpected variations in pulse signals were observed during critical situations. A few studies [53,54], however, reported that a tri-axial accelerometer embedded in a smart phone can be of relatively low quality and thus resulted in poorer fall detection performance compared to other standard commercially available accelerometers. Kau and Chen [55] conducted a study on smart phone-based fall detection using a tri-axial accelerometer and an electronic compass.

The recent introduction of the Microsoft Kinect has drawn the interests of many researchers back to vision-based fall detection. Based on the Kinect, Stone *et al.* [56] presented a two-state fall detection technique and validated the system on a large dataset collected in 13 apartments. The dataset consists of approximately 3339 days of continuous data, comprising 454 falls (with 445 falls performed by actors and nine natural falls). Ma *et al.* [57] presented a depth-based human fall detection technique, combining two computer vision techniques, *i.e.*, shape-based and learning-based classifiers. An insightful review on Kinect-based computer vision research and applications can be found in [58]. Table 1 shows examples of the existing ambient-sensor-based fall detection experiments.

## 3. Wearable Fall Detection Systems

This section focuses on a detailed overview of different aspects of fall detection, including sensor types and placement, subject details, activities of daily living (ADLs) and fall protocols, features, classification methods, and performance evaluation.

### 3.1. Sensor Placement

The wearable sensors that have been used in fall detection include tilt switches, accelerometers, gyroscopes, pressure sensors, and microphones. Among these, an accelerometer is considered one of the most effective and commonly used devices. Existing fall detection studies have been conducted with different sensor positions. With a single wearable sensor, the most common placement is the wearer's waist [32,38,46,65–77]. Other positions are the wrist [50,78–82], head [83], neck [84], trunk [85–88], chest [89,90], back [91–93], shoulder [94], armpit [95], ear [96,97], thigh [98], or foot [41,99]. Multiple sensors are sometimes used to enhance the fall detection algorithms [49,50,63,80,100–105] or to search for an effective sensor placement [35,48,49,78,79,81,82,89,106,107]. The devices are usually placed both on the upper body (*i.e.*, head, neck, chest, trunk, and waist) and lower body (*i.e.*, thigh, angle, and foot). The most common position on the lower body part is the thigh, which is usually combined with the chest/trunk [49,50,78,100–102,104,107] or waist [49,50,80,103]. Some studies, however, placed sensors only on the upper body [35,79,81,107,108].

**Figure 2.**
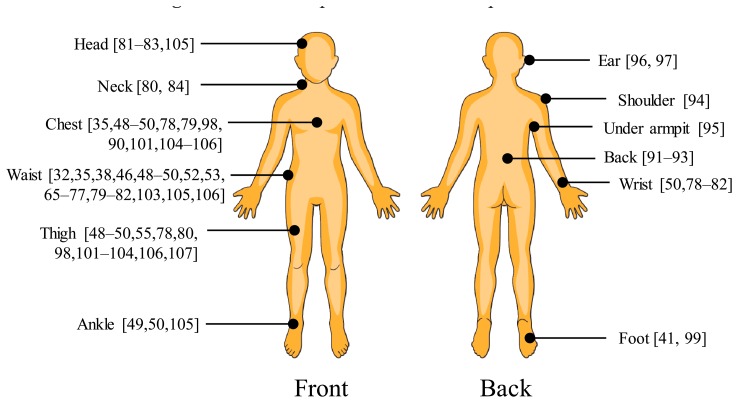
Different positions for sensor placement.

Several studies on optimal sensor placement have been conducted. Kangas *et al.* [81] studied fall detection by placing accelerometers on the subject's head, waist, and wrist. It was reported that the waist and head were efficient positions, while the wrist was not. Bourke *et al.* [107] placed sensors on the trunk and thigh, and reported the trunk as the better position. Bagnasco *et al.* [79] placed sensors on the waist, chest, and wrist, and reported the chest as the optimal position. Gjoreski *et al.* [49] compared sensors placed at the chest, waist, right ankle, and right thigh, and on the other hand, reported the waist as the optimal position. Dai *et al.* [48] conducted an experiment with smart phones placed on the subject's chest, waist, and thigh. The waist was also reported as the optimal placement. With a similar experiment setting, Fang *et al.* [106] reported that better performance was achieved when the sensor was placed near the center of mass. The results showed that the chest was the optimal position, but placement on the waist was recommended as it was more comfortable. Figure 2 summarizes the different sensor positions used in existing fall detection experiments.

### 3.2. Experimental Protocols

Most fall detection algorithms are designed based on datasets containing a mixture of ADLs (including fall-like activities) and simulated falls. The summaries of ADLs and simulated fall protocols used in previous fall detection studies are given in Tables 2 and 3, respectively.

The common ADLs include standing, walking (level), walking (up/down stairs), running/jogging, jumping, sitting down on chair, getting up from chair, lying down on bed, getting up from bed, and picking up object from floor. Other ADLs used in few studies and are not listed in Table 2 include ice skating [52], answering phone [53], waving phone [55], having chest pain [62], climbing into bed [46], hand shaking [79], having headache [62], hopping [38], jumping into bed [50], rotating in chair [38], sitting on or getting up from different types of chairs (*i.e.*, armchair, kitchen chair, stool) [88,89,107], and getting in and out of bathtub [29]. Some studies also include in their protocols transition activities, such as sit-stand, stand-sit [67], sit-lie, lie-sit [72,85,90,103], stand-walk [90], and walk-turn-walk.

In addition to Table 3, other types of falls include backward to lateral fall, forward fall with forward arm protection [44], faint [62,105], fall forward with 90° turn, fall while picking up something, collapse [96], and fall vertically [71]. Aziz *et al.* [105] involved different loss of balance gestures in their fall protocols, for example, while descending from standing to sitting, while rising from sitting to standing, while turning, and while reaching. In [81], Kangas *et al.* asked subjects to perform forward and lateral falls with a simulated missing step (*i.e.*, when stepping down from a platform). Gjoreski *et al.* [49] included slow falls with an attempt to hold onto the furniture in their data collection protocol. Brown [74] divided falls into simple fall (fall end up lying) and complex fall (fall end up with a vertical posture). Chen *et al.* [65] and Wang *et al.* [97] divided postures before a fall into stand, sit-to-stand, stand-to-sit, walk, walk backward, stoop, jump, and lie on the bed. Anania *et al.* [87] divided falls into four types, *i.e.*, while resting, walking, running, and jumping.

Types of falls that are difficult to be classified are varied across different studies. Bianchi *et al.* [46] conducted a fall detection experiment with eight types of falls and eight types of ADLs and reported falls with recovery as the most difficult fall to detect. Kangas *et al.* [81] reported that among forward, backward, and sideward falls, backward fall was most difficult to detect. Tolkiehn *et al.* [76] found that out of 13 types of falls, forward falls onto the knees were the most difficult to classify.

### 3.3. Features

A feature extraction module plays an important role in fall detection. Its goal is to extract parameters which represent discriminative information, such as body orientation, mechanical vibration, impact, activities, and/or velocity profile, to be used as input of a classification model.

Table 4 provides a list of features used in the existing fall detection experiments described in Table 5. The features are extracted from an accelerometer (denoted by *a*), a gyroscope (denoted by ω), or a pressure sensor (denoted by *p*). Most of the features are extracted from acceleration signals. Mean, standard deviation, sum vector magnitude, and tilt angles are simple, yet informative, features commonly used in existing fall detection experiments. Generic equations such as mean, standard deviation and variance can be applied to any motion sensors and therefore the inputs of the equations are denoted by *x*.

Mean (F1) and standard deviation (F2) can be calculated along each of the three axes (*x*, *y*, and *z*). Means are informative for detection of static activities (e.g., lying, sitting, and standing), while standard deviations are informative for distinguishing between static and dynamic activities and for classifying dynamic activities (e.g., walking, running, and jumping). Others informative features for classification of static activities include the angle between the device and ground (F20, F26) and the angle between the device and the gravitational vector (F22, F23, F24, F29, F32, F33). The later can be represented in various forms, namely, tilt angle (F23), inclination angle (F24), orientation angle (F29), sagittal angle (F32), lateral angle (F33), and device orientation change (F27). To calculate the angle between the device and the gravitational vector, some studies used the Earth's standard acceleration due to gravity (*g*) [67,104] or the sum vector magnitude (F5) equal to 1*g* (assuming no movement in a static activity) [46,49] in their equations. During static activities, the motion signals measured from a device are quite stable, and therefore the standard deviation is lower than those of static activities. Besides standard deviation, several studies used signal magnitude area (F10) to distinguish between static and dynamic activities [46,72,90,91].

Sum vector magnitude (F5) has been used in most studies for detecting abnormal signals. The highest peak during the sudden change of this value is used to indicate a fall [32,38,46,48–50,53,65,67–70,72–75,77–82,84–86,89–91,93,96,97,101,102,104,106,107]. In [85], F5 was used to indicate the free-fall state (*i.e.*, vertical speed increases linearly with time due to gravity). Using only F5 alone, however, is not sufficient for accurate fall detection since jumping at once also generates a sudden change of this feature which can trigger a false alarm. To solve this problem, the activity that follows the peak is sometimes used. For example, if the sudden change is followed by standing, then it is not a fall. Velocity during fall (F12, F13, F14), vertical acceleration (F15), or differential pressure (F42) are sometimes used in addition to F5 to achieve higher detection accuracy [32,46,65,67,70,97]. In [83], a multiple regression equation of the absolute peak acceleration values in the movement and the horizontal directions (F43) was used to detect falls.

F37–F41 are calculated using a gyroscope. Similar to F5 of an accelerometer, the resultant angular velocity (F40) can be used to identify possible falls using a gyroscope. For fall detection, resultant angular acceleration (F39) and resultant change in trunk angle (F41) were used in [88]. To derive F39 and F41, trunk angle (F37) and trunk angular acceleration (F38) are first calculated. More accurate device orientation, particularly during dynamic motions, can be obtained by combining signals from different types of motion sensors. In [55], an electronic compass was used in conjunction with a tri-axial accelerometer to estimate the orientation of a smart phone. F44–F50 are accelerometer-based features used in existing studies with no equation provided. They are referred to in Table 5 and therefore are also listed for completeness.

### 3.4. Classification and Evaluation

Methods for fall classification can be broadly categorized into threshold-based, rule-based and machine learning approaches. Simple thresholding, however, is not suitable to detect different types of falls. Most automatic fall detection systems rely on the rule-based approach [38,46,47,50,65,67–74,76,78–81,83–94,96–98,102–104,106]. The main concept is to construct a set of rules for detecting ADLs or falls based on thresholds of one or more features. Commonly used features include mean, standard deviation, sum of vector magnitudes, and tilt angle. In [109], information regarding user's activities before and after a fall was also used to enhance the detection accuracy.

Several fall detection systems are based on or partly based on machine learning. Examples of algorithms used in fall detection experiments include Decision Tree (DT) [49,101], Naïve Bayes (NB) [49,50], Hidden Markov Model (HMM) [62,66], Gaussian Mixture Model (GMM) [66], k-Nearest Neighbor (kNN) [110], k-mean [50], Support Vector Machine (SVM) [41,49,61,99], Fuzzy Inference System (FIS) [77], and Artificial Neural Network (ANN) [77]. Gjoreski *et al.* [49] used machine learning algorithms, such as J48, Naïve Bayes, random forest, and SVMs, in their body posture recognition module. Li *et al.* [50] applied k-mean, Naïve Bayes, entropy discretization, and regression for the same purpose. In [101], a decision tree (C4.5) constructed from sum vector magnitude and raw data was used to distinguish between falls and ADLs. Majority filtering was applied over outputs within a non-overlap window and a fall can be identified when the majority of the sample windows reach a consensus. Zhang *et al.* [110] discriminated between falls and non-fall activities using kNN with features extracted from their proposed Non-negative Matrix Factorization (NMF) algorithm.

Analyzing relationships between falls and resulting injuries, as well as patterns and long-term trends of fall conditions, can potentially be useful for establishing efficient fall prevention strategies. Although different types of stimulated falls have been addressed in many studies, only few studies have focused on fall type classification. Tolkiehn *et al.* [76] presented a fall detection algorithm for classifying falls into three directions (forward, backward, and left/right). Hseih *et al.* [70] proposed an algorithm that can classify eight different fall types based on tri-axial acceleration. Fall directions (forward, backward, left, and right) were first determined, followed by identifying the impact parts. Hands with elbow or hip were considered as the impact parts for falling forward left and right, back of body or hip for falling backward, and hands with elbow or knees for falling forward. Aziz *et al.* [105] proposed a system for detecting the cause of a fall, namely, slips, trips, and other types of imbalance.

To evaluate the practicality of the system, the classification model should be evaluated on datasets acquired from both young and elderly subjects. Elderly people were normally asked to perform only ADLs while young subjects were asked to perform both ADLs and simulated falls. In existing studies, the age of young subjects are between 19 and 50 years, and that of elderly subjects are between 55 and 83 years. Most fall experiments rely on only a dataset performed by young healthy subjects. However, several studies included ADLs protocols performed by elderly subjects to evaluate the false positive rates of the proposed algorithms [67,68,85,88,96,107]. The analysis of fall signals acquired from elderly subjects can be found in [69,92]. In [91], both young and elderly subjects were asked to walk on a treadmill equipped with a safety harness to protect actual falls. To induce a free fall phase, each subject walked on the treadmill for 2 min without and with obstacles randomly placed on the treadmill every few seconds.

Some studies involved more than one datasets. Tamura *et al.* [71] collected three different datasets for the development of an airbag fall prevention algorithm. To investigate the appropriate time for inflating the airbag, a dataset was acquired from fourteen young subjects while performing stimulated fall protocols without wearing the airbag (Test A). To evaluate the false positive rate, another dataset was acquired from nine physiotherapists while performing only ADL protocols (Test B). To evaluate the performance of the device, four young subjects were asked to perform a simple backward fall while wearing the prototype device with the airbag (Test C). Bianchi *et al.* [46] conducted three different experiment protocols in different environments, *i.e.*, indoor, outdoor, and both indoor and outdoor. The obtained datasets were used for algorithm development and for evaluating the false negative rate and the false positive rate. To our knowledge, there exists only one available online fall database acquired using a vision-based device [111] and there is still no publicly available database to support wearable-sensor-based fall detection research.

In addition to the number of subjects, different types of falls and ADLs involved in the experiments, as well as their complexity, should also be considered in performance evaluation of an algorithm. Most studies evaluate their models using one or more of the three performance measures calculated from a confusion matrix, namely, sensitivity, specificity, and accuracy. Sensitivity reflects the ability of the system in detecting falls, specificity reflects the ability of the system in detecting ADLs, and accuracy is the overall ability of the system in detecting both falls and ADLs. Table 5 shows a summary of existing wearable-sensor-based fall detection experiments. The listed experiment attributes include authors, sensor types and placements, subject details (number of subjects, age range and gender), number of falls and ADLs, features (with reference to Table 4), classification methods, and performance. Types of sensors and subjects are represented using graphical icons. The stars on sensor icons indicate that sensors at different positions were combinedly used at the same time. Their absence indicates that the subject wore the device (possibly with multiple sensors) at one position at a time while performing the experiment protocols. Some of these studies can also send a real-time alert when a fall is detected [38,41,46,47,49,50,65,69–77,79,83–87,89,90,96–99,101–104,106,112].

## 4. Fall Detection Products

There exist several fall detection products currently available in the market. Most of them are wearable devices with ease-of-use designs. The most common device placement position is the waist, but some devices are designed to be placed on the wrist, or around the neck. Most products use an acceleration sensor and lithium battery, which has a maximum lifetime of up to 2 years. They usually come with an alarm button allowing a manual call for help when a fall is not automatically detected. Table 6 illustrates some examples of existing wearable fall detection products.

## 5. Future Trends

As guidelines for future fall monitoring research, this section presents the trends of fall detection devices, as well as addresses the difference between simulated and real world fall conditions. To reduce the causes of deaths and to improve the quality of life in elderly population, not only fall detection but also fall-risk assessment and fall prevention should be investigated.

### 5.1. Devices

The number of fall studies by using camera-based and wearable devices is still increasing [25]. Using a camera seems to be a reliable approach for fall detection, a camera-based system can provide a high percentage of sensitivity and specificity [29,35]. However, the major disadvantages of this approach are the complex setup, area constraints and the lack of privacy. Wearable sensors are therefore still more popular than cameras in fall monitoring. Furthermore, most wearable sensors are now embedded in smartphones. The advantages of using a smartphone are cost effectiveness, usability in both indoor and outdoor environments, and ability to track a user using the GPS module in the phone [53].

### 5.2. Real World Falls

The datasets for fall detection in most existing studies were acquired from young healthy subjects both during simulated falls and ADLs. When performing simulated falls, the subjects are often instructed to fall directly onto a mattress. However, in real world conditions, fallers may try to break their falls with their hands before collapsing on the ground or may fall slowly [46]. The impact surface can also be hard. These conditions are different from simulated falls in laboratory settings. So the generalization of the methods developed needs to be carefully assessed.

Most existing fall detection systems can detect only falls, while other types of information such as direction, time-to-fall, lying fall period, location, and fall status are not yet comprehensively addressed. Such additional information should be collected and sent to hospitals or family members when falls occur. Time-to-fall is a useful piece of information when one wants to develop a fall prevention tool such as a wearable airbag [71]. Accurate localization can help an assistant to reach a faller in a timely manner. The lying fall period, the direction of fall, and the faller status can help to estimate the severity of a fall so that appropriate medical support can be dispatched without delay.

### 5.3. Fall Risk Assessment and Fall Prevention

Instead of detecting adverse events and reacting in a timely manner, an even better solution would be to take a proactive approach by understanding fall risk factors and developing fall prevention mechanisms. The first step of fall prevention is to understand the risk of falling. In 2000, Brauer *et al.* [131] reported the results of their investigation on the ability of various laboratory measures and clinical tests of postural balance in predicting falls in one hundred elderly female population within a period of 6 months after the tests. Postural muscle timing, movement speed, and the center of pressure motion were recorded during one or more laboratory tasks (*i.e.*, reaction-time step task, a limit of stability, and a quiet stance balance task). Four clinical tasks include the Berg Balance Scale [132], the Functional Reach Test [133], the Lateral Reach Test [134], and the Step-Up Test [135]. Based on logistic regression models, the study reported a fall prediction rate of 77%, with the sensitivity of only 51%. The results indicate that there is still plenty of room for improvement in this research area.

Examples of popular measures for fall risk assessment include STRATIFY (St. Thomas's risk assessment tool in falling elderly inpatient) [136], TUG (Time up & go) [137], Barthel index [138], and TGBA (Tinetti Gait and Balance Assessment) [139]. TUG score, for example, is the easiest and most commonly used measure. For movement evaluation, the elderly people are asked to perform a sequence of activities including stand up from a chair, walk with normal speed for 3 meters, turn around, walk back to the chair and sit down on the chair. Time to completion is recorded and if it takes longer than 14 s, the risk of falling is considered high. King *et al.* [140] illustrated the use of a miniaturized wireless e-AR (ear-worn activity recognition) in fall risk assessment and concluded that the sensor was able to detect several features from TUG assessments. Ghasemzadeh *et al.* [141] presented a physiological monitoring system based on tri-axial acceleration and EMG sensors. The acceleration and muscle activity signals were collected while subjects were standing on a half circle ball, and were processed through a statistical machine learning technique to assess postural balance. Jiang *et al.* [142] proposed a walking model for fall risk assessment based on an accelerometer placed on the subject's waist.

Recently, several studies have focused on preventing falls. Fukaya *et al.* [143] introduced the idea of launching a jacket-worn airbag which will be inflated to protect the head and buttocks based on tri-axial acceleration signals. It was designed to protect falls from height, falls from wheelchair overturn, and falls on the same level. However, the system was validated on a dummy. Tamura *et al.* [71] developed a jacket-worn airbag to protect the head, neck, hip, and thigh based on a tri-axial accelerometer and a gyroscope. Guangyi *et al.* [144] developed a belt-worn airbag using a tri-axial accelerometer and a gyroscope.

## 6. Conclusions and Future Directions

This paper presents a comprehensive review of automatic fall monitoring. In general, automatic fall monitoring can be categorized as wearable-sensor-based, ambient-sensor-based or combined-sensor-based approaches. In several studies, cameras are employed as a type of ambient sensors. Although video cameras can provide sufficient information for patient monitoring, field-of-view constraints, lighting condition, camera positioning and lack of privacy are still major limitations. Wearable sensors have advantages over ambient sensors in terms of mobility, ease of installation, coverage areas of usage and less privacy constraint. They are thus a relatively practical and cost-effective solution to support independent living for elderly people. This study provides a timeline review of the alternative technologies and particularly focuses on wearable sensor-based fall monitoring.

To establish a fair comparison across different research studies and reflect the state-of-the-art of the technology, this paper provides an in-depth review of important aspects of existing fall detection experiments, including sensor types and positions, data collection protocols, subject groups, feature extractors, classification methods, and performance measures. Apart from a detailed summary of existing research studies, we also present an overview of available commercial products and future research directions of automatic fall monitoring. Commercial products are described and compared based on size, weight, sensor type, battery, transmission range, and features. The use of mobile devices, including smart phones, as the hardware platform is becoming increasingly popular. They can be used not only for motion sensing, but also for storing profiles, communication and detecting users' locations. In addition to fall alerts, a user's location or co-location is also another important information that allows timely assistance and intervention. Although smart phones are already being carried by users on a daily basis and seem to be a promising solution for fall monitoring, there still exist many issues to be addressed, *i.e.*, sensor fixation, varied sensor quality, and battery lifetime. At the moment, dedicated-designs of wearable fall sensors with constraint positioning are still relatively better in terms of battery lifetime and achievable accuracy.

In terms of sensor placement, several studies have reported that the best accuracy can be achieved when sensors were placed near the center of mass. However, in commercial product designs, user acceptance and usability must also be considered, perhaps with a tradeoff of accuracy. Assessment of devices' accuracy and practical values is indeed difficult. This is due to the fact that there still exists a very limited amount of natural falls datasets (particularly in elderly people) and the absence of publicly available real-life datasets for benchmarking. In many real scenarios, elderly people also tend to fall slowly, which may not be captured by existing systems. Beyond automatic fall detection, there has been a recent rapid surge of interests in fall risk assessment and fall prevention, aiming towards a proactive prevention approach of elderly care. These indicate several problems yet to be addressed by the research community.

## Figures and Tables

**Table 1.**
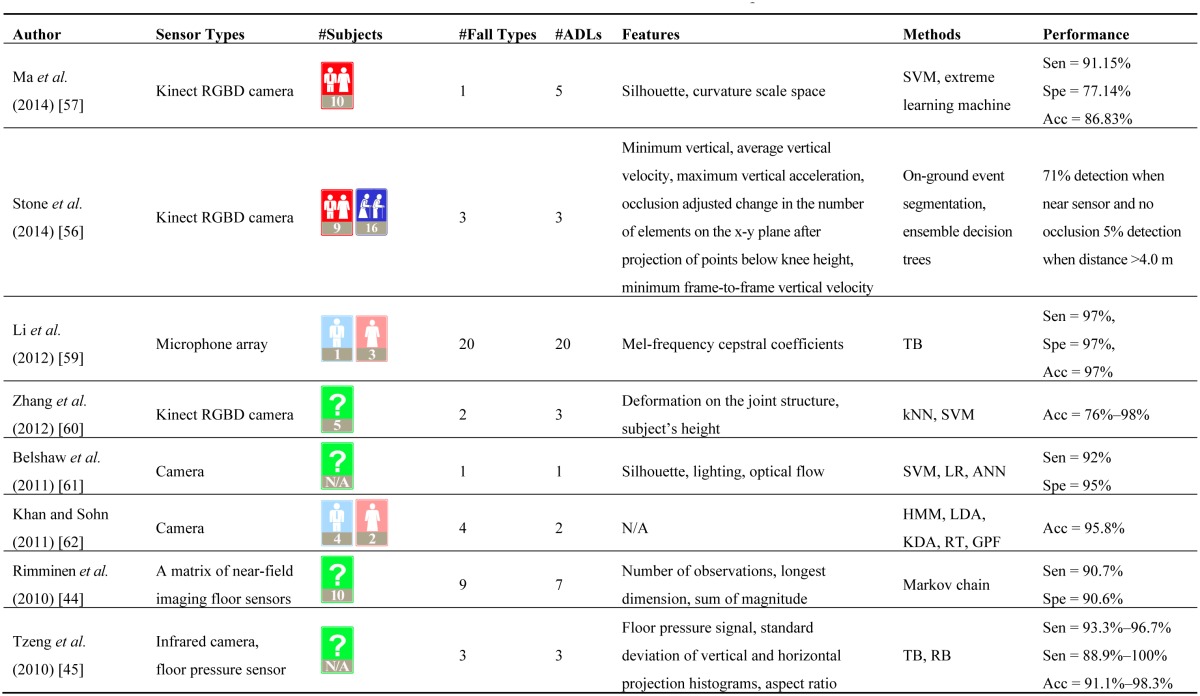

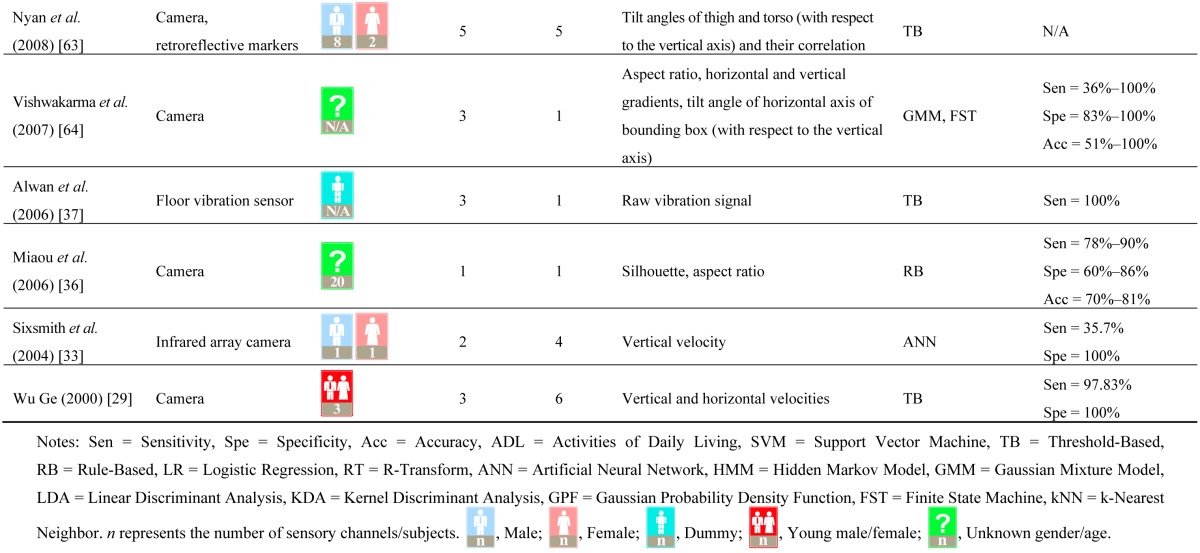
Ambient-sensor-based fall detection experiments.

**Table 2.** Activities of Daily Living (ADL) protocols.

**Reference**	**Collapse into chair**	**Get in/out of car seat**	**Get up from bed**	**Get up from chair**	**Get up from stool**	**Jump**	**Pick up object from floor**	**Lean backward**	**Lean forward**	**Lying**	**Lying down on bed**	**Lying left**	**Lying on back**	**Lying on stomach**	**Lying right**	**Rest against a wall**	**Running/jogging**	**Search for object on floor**	**Sitting**	**Sitting on bed**	**Sitting on chair**	**Sitting on chair (fast)**	**Sitting on floor**	**Sitting on stool**	**Sprinting**	**Squatting**	**Standing**	**Stooping/bending**	**Stretching**	**Stumble**	**Take a lift down**	**Take a lift up**	**Throwing oneself on back on a bed**	**Walking (level)**	**Walking downstairs**	**Walking upstairs**	**Walking with luggage**
[55]						•											•		•															•	•	•	
[75]			•	•													•			•	•					•							•	•			
[84]										•							•				•		•				•	•						•	•	•	
[106]																			•								•							•			
[69]			•	•			•													•	•													•	•	•	
[79]			•	•				•	•		•										•	•												•			
[49]											•							•			•	•															
[93]				•																	•													•			
[53]							•																											•	•	•	
[50]						•				•							•		•			•					•			•				•			
[98]				•													•				•				•									•	•	•	
[101]											•										•						•							•			
[76]	•		•	•		•	•				•					•					•							•			•	•			•	•	
[66]						•											•				•						•							•			
[46]	•					•	•														•							•			•				•		
[65]						•					•						•			•								•						•	•	•	
[48]																	•		•								•							•			
[83]				•							•						•				•													•	•	•	•
[85]																												•						•			
[80]										•									•								•							•			
[102]										•									•									•									
[68]			•	•			•				•										•													•	•	•	
[104]						•					•											•								•				•	•	•	
[71]			•										•								•													•			
[86]				•																	•					•								•			
[87]						•											•																	•			
[89]											•									•	•													•			
[88]		•	•	•	•						•										•			•										•			
[78]												•	•	•	•				•								•							•			
[41]																		•																•			
[81]							•																											•	•	•	
[103]										•																									•	•	
[97]										•									•								•							•	•	•	
[107]		•	•	•																•	•													•			
[99]																	•																	•			
[82]							•																											•	•	•	
[38]																			•								•							•	•	•	
[72]										•																								•			
[35]			•	•			•													•	•													•			
[73]																			•															•			
[74]						•	•										•		•										•					•		•	
[96]													•				•				•													•		•	

**Table 3.** Simulated fall protocols.

**Reference**	**Backward fall (BW)**	**BW (impact on back)**	**BW (end up sitting)**	**BW (end up lying)**	**BW (end up lying left/right)**	**BW (impact on hip)**	**BW (round back, knee flexion)**	**BW (attempt to get up)**	**BW (knee flexion)**	**BW (legs straight)**	**Collapse into bed**	**Fall**	**Fall against wall**	**Fall end up sitting**	**Fall from chair**	**Fall from bed**	**Fall on stairs**	**Fall left (impact on hands and elbows)**	**Fall to left (impact on hip)**	**Fall right (impact on hands and elbows)**	**Fall right (impact on hip)**	**Fall with recovery (then walking)**	**Fall with recovery (then standing)**	**Forward fall (FW)**	**FW knee flexion**	**FW (impact on hands and elbows)**	**FW (impact on knee)**	**FW on knee**	**FW end up lying**	**FW (attempt to get up)**	**FW (attempt to break fall)**	**FW (knee flexion)**	**FW (legs straight)**	**Lateral fall**	**Fall left**	**Falls left (knee flexion)**	**Falls left (legs straight)**	**Fall right**	**Falls right (knee flexion)**	**Falls right (legs straight)**	**Slip**	**Syncope**	**Trip**
[55]												•																															
[75]	•														•	•								•										•									
[84]	•																•							•											•			•					
[52]	•																							•											•			•					
[106]												•																															
[69]														•		•																		•							•	•	•
[105]																																									•		•
[79]	•																							•											•			•					
[49]																																											•
[70]		•				•												•	•	•	•					•	•																
[93]																								•	•										•			•					
[53]	•																							•											•			•					
[50]	•												•											•											•			•					
[98]	•										•													•																			
[101]	•																							•											•			•					
[76]			•	•				•			•				•	•						•	•					•	•	•					•			•					
[66]																								•																			
[46]				•									•									•	•						•	•	•			•									
[67]									•	•																						•	•			•	•		•	•			
[48]	•																							•										•									
[83]	•																							•										•									•
[85]	•																							•											•			•					
[92]	•																																										
[80]												•																															
[102]	•																							•										•									
[77]	•											•												•										•									
[68]														•		•																		•							•	•	•
[104]	•																																										
[71]	•													•		•								•											•			•					
[86]																								•										•									
[89]									•	•																						•	•			•	•		•	•			
[88]									•	•																						•	•			•	•		•	•			
[78]	•																							•											•			•					
[41]												•																															
[81]	•		•		•	•	•							•										•										•									
[103]	•																							•											•			•					
[107]									•	•																						•	•			•	•		•	•			
[99]												•																															
[90]	•																							•											•			•					
[82]	•																							•										•									
[38]	•																							•											•			•					
[35]	•																																		•			•					
[73]	•																							•																			
[96]	•					•							•											•																			
[32]	•																							•										•									

**Table 4.** Features for fall detection experiments.

**No.**	**Feature**	**Equation**
F1	Mean	$\mu =\frac{1}{N}\sum_{i=1}^{N}x_{i}$
F2	Standard deviation	$\sigma =\sqrt{\frac{1}{N}\sum_{i=1}^{N}{\left(x_{i}-\mu \right)}^{2}}$
F3	Variance	$\sigma^{2}=\frac{1}{N}\sum_{i=1}^{N}{\left(x_{i}-\mu \right)}^{2}$
F4	Standard deviation magnitude	$\left|\sigma \right|=\sqrt{\sigma_{x}^{2}+\sigma_{y}^{2}+\sigma_{z}^{2}}$
F5	Sum vector magnitude	$\left|a\right|=\sqrt{a_{x}^{2}+a_{y}^{2}+a_{z}^{2}}$
F6	Sum vector on horizontal plane	${\left|a\right|}_{h}=\sqrt{a_{x}^{2}+a_{z}^{2}}$
F7	Standard deviation of sum vector magnitude	$\sigma_{\left|a\right|}=\sqrt{\frac{1}{N}\sum_{i=1}^{N}\left({\left|a\right|}_{i}-\mu_{\left|a\right|}\right)}$
F8	Difference between maximum and minimum values of sum vector magnitude	Δ|*a*|_max−min_ = max(|*a*|)−min(|*a*|)
F9	Root mean square of sum vector magnitude	${\left|a\right|}_{\text{r}ms}=\sqrt{\frac{1}{N}\sum_{i=1}^{N}{\left|a\right|}_{i}^{2}}$
F10	Signal magnitude area	$\text{SMA}=\frac{1}{t}\left(\int_{0}^{t}\left|a_{x}\left(t\right)\right|dt+\int_{0}^{t}\left|a_{y}\left(t\right)\right|dt+\int_{0}^{t}\left|a_{z}\left(t\right)\right|dt\right)$
F11	Activity signal magnitude area	$\text{ASMA}=\frac{1}{t_{2}-t_{1}}\left(\int_{t_{1}}^{t_{2}}\sqrt{a_{x}^{2}\left(t\right)+a_{y}^{2}\left(t\right)+a_{z}^{2}\left(t\right)dt}\right)$
F12	Reference velocity	$\upsilon_{\text{ref}}=\int_{t_{\text{rest}}}^{t_{\text{tilt}}}\left(\left|a\left(t\right)\right|-g\right)dt$
F13	Velocity	*υ* =∫(|*a*(*t*)| − *g*)*dt*
F14	Velocity (approximate)	$\upsilon_{2}=\sqrt{{\left(\int a_{x}\left(t\right)dt\right)}^{2}+{\left(\int a_{y}\left(t\right)dt\right)}^{2}+{\left(\int a_{z}\left(t\right)dt\right)}^{2}}-\int \text{gdt}$
F15	Vertical acceleration	$a_{v}=\frac{1}{2g}\left({\left|a\right|}^{2}-{\left|a\right|}_{\text{dynamic}}^{2}-g^{2}\right)$
F16	Maximum vertical acceleration	(*a_v_*)_max_=max(*a_z_*)
F17	Average acceleration change	$\bar{\Delta \left|a\right|}=\frac{1}{T_{n}-T_{0}}\sum_{i=0}^{n-1}\left(\left|a\left(t+1\right)\right|-\left|a\left(t\right)\right|\right)$
F18	Overall acceleration value	a*_overall_* = E[||a|^2^−E[|a|^2^]|]
F19	Acceleration amplitude at absolute vertical direction	|*a_v_*|=|*a_x_* sin^θ^*_z_* + *a_y_* sin^θ^*_y_* −*a_z_* cos^θ^*_y_* cos^θ^*_z_*|
F20	Angle between device and ground	ρ*_x_* = sin(*a_x_*), ρ*_y_* = sin(*a_y_*), ρ*_z_* = sin(*a_z_*)
F21	Angle between device and gravity	$\theta_{x}=\sin^{-1}\left(\frac{a_{x}}{g}\right),\theta_{y}=\sin^{-1}\left(\frac{a_{y}}{g}\right)$
F22	Angle between z axis and vertical (with respect to the gravity)	$\theta =\text{atan2}\left(\sqrt{{\text{a}}_{x}^{2}+{\text{a}}_{y}^{2}},{\text{a}}_{z}\right)$
F23	Tilt angle (with respect to the gravity)	θ=cos^−1^(a*_Z_*)
F24	Inclination angle (with respect to the gravity)	$\theta =cos^{-1}\left(\frac{a_{z}}{g}\right)$
F25	Posture (inclination angle with respect to the gravity, calculated using dot-product method)	$\theta \left(t\right)=\cos^{-1}\left(\frac{{\vec{g}}_{s}\left(t\right)\cdot {\vec{g}}_{r}}{\left|{\vec{g}}_{s}\left(t\right)\right|\cdot \left|{\vec{g}}_{r}\right|}\right)\left(\frac{180}{\pi }\right)$
F26	Orientation of person's trunk (with respect to the ground)	$\rho =tan^{-1}\left(\frac{\sqrt{a_{x}^{2}+a_{y}^{2}}}{a_{z}}\right)$
F27	Device orientation change	$\theta =cos^{-1}\left(\frac{a_{x}\mu_{x}+a_{y}\mu_{y}+a_{z}\mu_{z}}{\sqrt{\mu_{x}^{2}+\mu_{y}^{2}+\mu_{z}^{2}}.\sqrt{a_{x}^{2}+a_{y}^{2}+a_{z}^{2}}}\right)\left(\frac{180}{\pi }\right)$
F28	Orientation change	θ = **ā**(*t_b_*) **ā**(*t_a_*)
F29	Orientation angle (with respect to the gravity)	$\theta =cos^{-1}\left(\frac{a_{z}}{\sqrt{a_{x}^{2}+a_{y}^{2}+a_{z}^{2}}}\right)$
F30	Ratio between two consecutive angles	$\theta_{\text{ratio}}=\frac{\theta \left(t_{i}\right)}{\theta \left(t_{i+1}\right)}$
F31	Difference between two consecutive angles	Δθ=θ(*t_i_*_+1_) − θ(*t_i_*)	
F32	Sagittal angle (with respect to the gravity)	$\theta_{s}=-tan^{-1}\left(\frac{a_{y}}{a_{z}}\right)\left(\frac{180}{\pi }\right)$	
F33	Lateral angle (with respect to the gravity)	$\theta_{l}=tan^{-1}\left(\frac{a_{x}}{\sqrt{1-a_{x}^{2}}}\right)\left(\frac{180}{\pi }\right)$	
F34	Horizontal angle from *x*-axis in *xy*-plane	$\theta_{h}=tan^{-1}\left(\frac{a_{x}}{a_{y}}\right)$	
F35	Vertical angle from *x*-axis	$\theta_{v}=sin^{-1}\left(\frac{\sqrt{{a_{x}}^{2}+{a_{y}}^{2}}}{\left|a\right|}\right)=cos^{-1}\left(\frac{a_{z}}{\left|a\right|}\right)$	
F36	Jerk (rate of acceleration change)	$\frac{\Delta a}{\Delta t}=\frac{a_{x}\left(t_{i}\right)-a_{x}\left(t_{i-1}\right)}{0.001}$	
F37	Trunk angle	$\theta_{\text{pitch}}=\int_{t=-1.2s}^{t=0.5s}\omega_{\text{pitch}}\left(t\right)dt,\theta_{\text{roll}}=\int_{t=-1.2s}^{t=0.5s}\omega_{\text{roll}}\left(t\right)dt$	
F38	Trunk angular acceleration	$\alpha_{\text{pitch}}=\frac{d}{dt}{\left\{\omega_{\text{pitch}}\right\}}_{-0.5s}^{0.5s},\alpha_{\text{roll}}=\frac{d}{dt}{\left\{\omega_{\text{roll}}\right\}}_{-0.5s}^{0.5s}$	
F39	Resultant angular acceleration	$\alpha_{r}=\sqrt{\alpha_{\text{pitch}}^{2}+\alpha_{\text{roll}}^{2}}$	
F40	Resultant angular velocity	$\omega_{\text{r}}=\sqrt{\omega_{\text{pitch}}^{2}+\omega_{\text{roll}}^{2}}$	
F41	Resultant change in trunk angle	$\theta_{\text{r}}=\sqrt{\theta_{\text{pitch}}^{2}+\theta_{\text{roll}}^{2}}$	
F42	Differential pressure	$\Delta p_{i}=\frac{t}{2}\left[\left(\sum_{k=i}^{k=i+(2/t)}p_{k}-\sum_{k=i-(t/2)}^{k=i}p_{k}\right)\right]$	
F43	Multiple regression equation	*Y* = −0.139 + 0.0195*X*_1_ + 0.0163*X*_2_	
F44	Maximum acceleration derivative	N/A	
F45	Maximum peak-to-peak acceleration amplitude	N/A	
F46	Maximum peak-to-peak acceleration derivative	N/A	
F47	Timestamp of falling body to be at rest	N/A	
F48	Timestamp of body's initial contact to ground	N/A	
F49	Time difference between when inclination angle exceed a critical angle and inclination velocity has local maximum above a threshold	N/A	
F50	Variation of ^|^*^a^*^|^ around 1 *g*	N/A	

Notes: *N* = number of data samples, *x* = observation, *i* = index of data sample, *g* = 9.81 ms^−2^, a*_x_*, a*_y_*, *a_z_*, are acceleration values along the *x*- (sideward), *y*-(forward), and *z*- (upward) axes, respectively, **a_¯_** = average acceleration vector, *t_a_* = time before fall, *t_b_* = time after fall, *t_tilt_* = time when body tilts, *t_rest_* = initial time when body is at rest, *g⃗_s_* = gravity vector estimated with respect to the body segment, *g⃗_r_* = the reference gravitational vector, *X*_1_ = the absolute peak value in the movement direction, *X*_2_ = the absolute peak value in the horizontal direction.

**Table 5.**
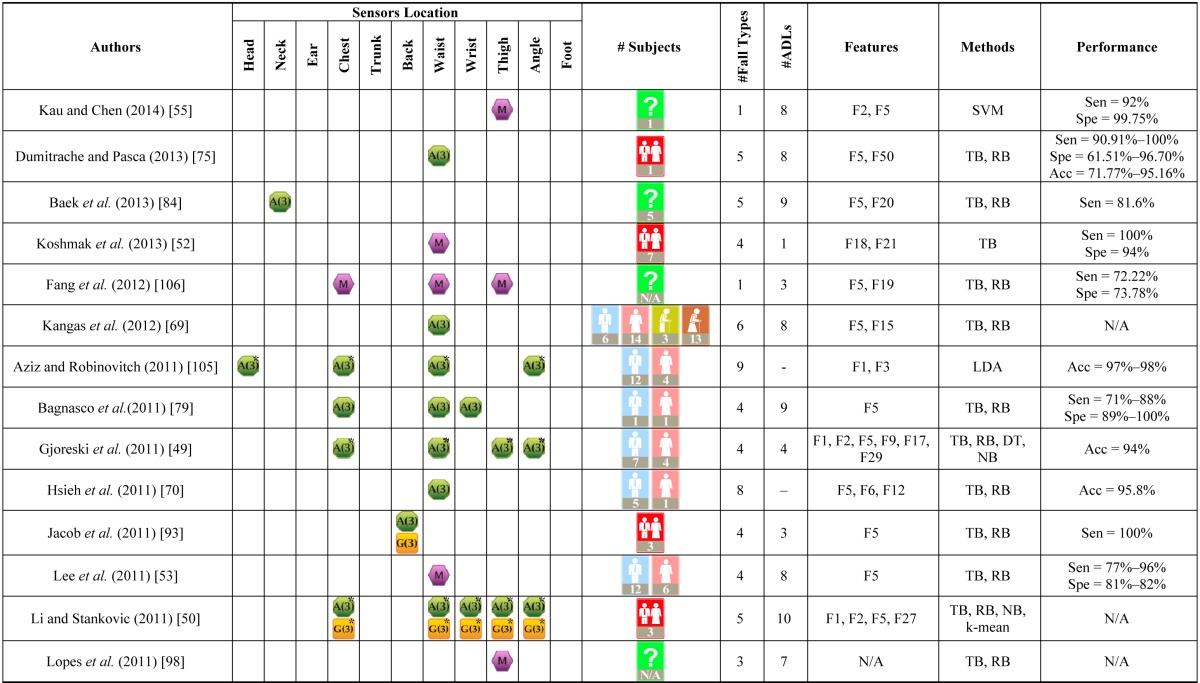

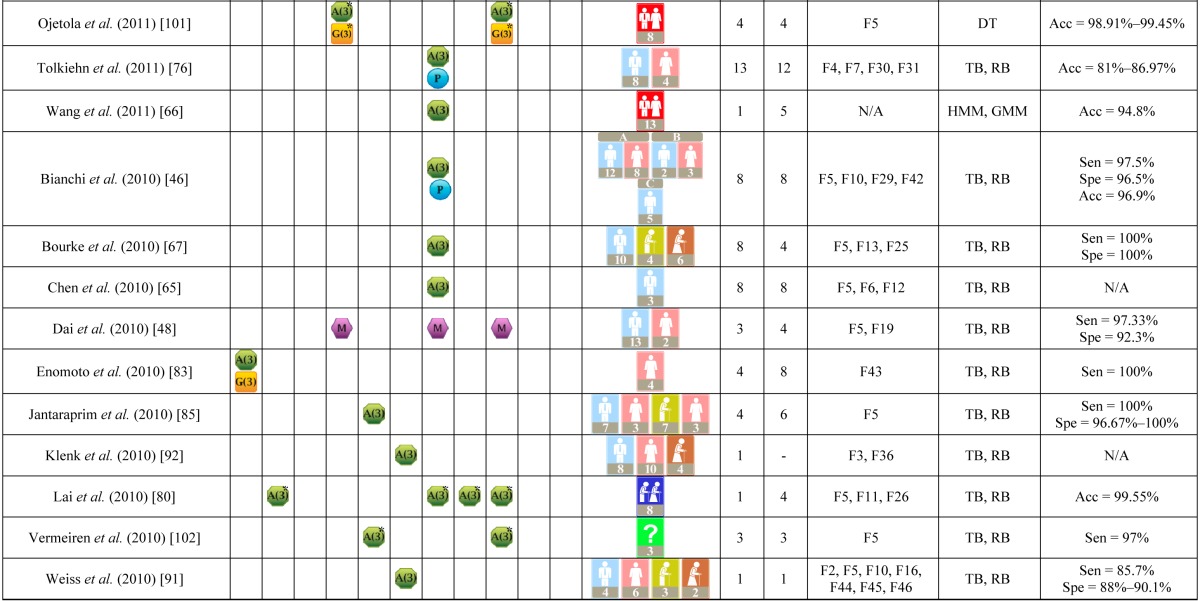

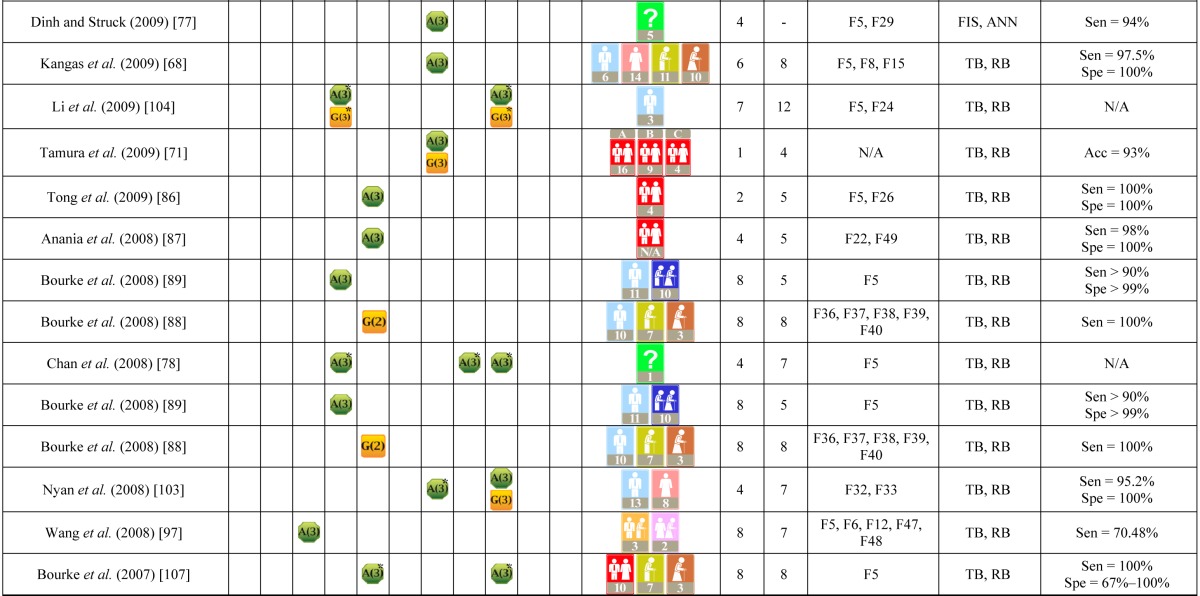

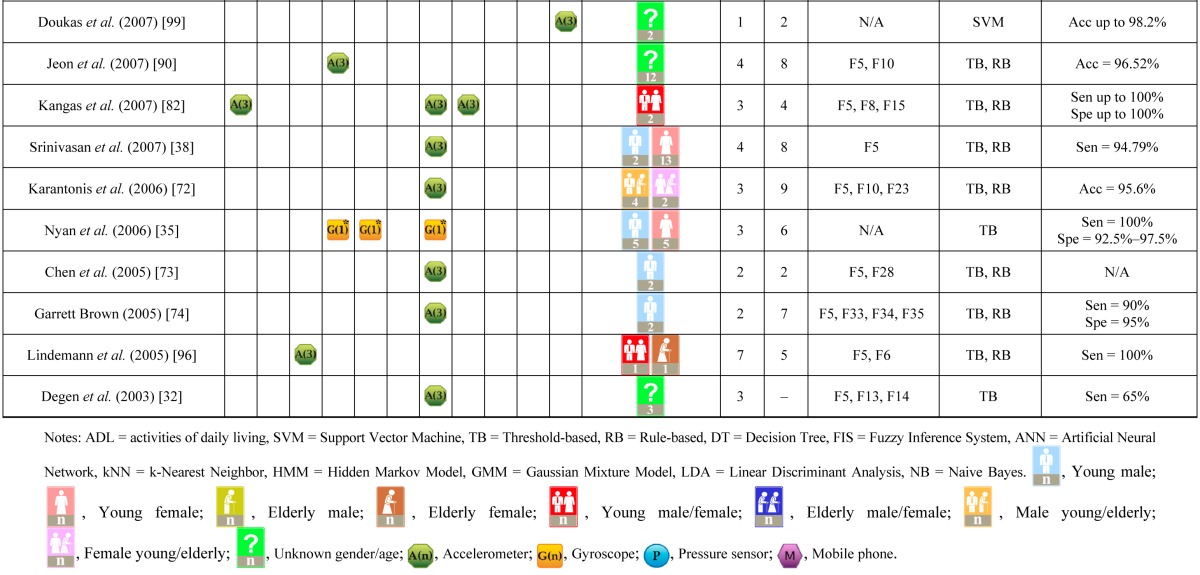
Existing fall detection experiments.

**Table 6.** Wearable fall detection products.

**Name**	**SENSO [113]**	**MySOS Mandown [114]**	**SensorBand [115]**	**Badge-iT Fall Detector [116]**	**Fall detector MCT-241MD PERS [117]**	**VitalBase [118]**	**Climax Fall Sensor [119]**	**Tunstall iVi Pendant [120]**	**Oval Fall Detector [121]**	**AFrame the Watch [122]**	**Philips Lifeline's AutoAlert Pendant [123]**	**Tunstall Fall Detector [124]**	**Tynetec Fall Detector [125]**	**Blue Alert Fall Detection Sensor [126]**	**CSEM Wrist Fall Detection[127]**	**Tynetec Wrist Worn Fall Detector [128]**	**onAll Chest Strap [129]**	**70 Degree Verso Fall Detector [130]**
Size (mm)	65 × 43 × 13	70 × 42 × 17	51 × 32 × 17	35 × 60	67 × 41 × 21	37 × 12	58.6 × 42 × 19	58 × 38 × 14	58.6 × 42 × 19	N/A	66 × 30 × 17	75 × 53 × 28	85 × 56 × 20	N/A	N/A	57 × 34 × 16	N/A	N/A
Weight (g)	N/A	43	N/A	N/A	N/A	35	N/A	25	N/A	N/A	32	75	68	N/A	N/A	23	N/A	N/A
Sensor Type	N/A	3D accelerometer	3D accelerometer	N/A	N/A	N/A	N/A	N/A	N/A	N/A	N/A	N/A	2D accelerometer	N/A	3D accelerometer	2D accelerometer/Pressure	N/A	N/A
Placement	Waist	Neck/Waist	Waist	Waist	N/A	Wrist	Neck	Neck/Waist/Chest	Neck	Wrist	Neck	Waist	Waist/Neck	Waist	Wrist	Neck	Chest	Neck/Waist
Battery Type	Lithium Polymer	Lithium-ion	Lithium	Lithium	Lithium	Lithium	Lithium	Lithium	Lithium	N/A	N/A	6V Duracell PX28L	Lithium	N/A	Lithium-Polymer	N/A	N/A	N/A
Battery Life	Up to 2 years	25 h	1 year	N/A	N/A	2 years	2 years	12 months	2 years	N/A	18 months	6 months	1 year	N/A	15 days to one month	2–3 years	10 h	3 years depending on usage
Range (m)	N/A	N/A	N/A	450	N/A	200	130–160	50	130	N/A	N/A	N/A	75	N/A	N/A	N/A	N/A	100
Features	Automatic fall detection Send emergency text from user phone to emergency contact(s) Fit with emergency button Suitable for all levels of user mobility Usable in and away from home No need for costly call center support Battery life: 2 weeks when fully charged or 2 years on transmission of 1 alarm a day	Automatic fall detection Dual band: 900/1800 MHz with GPRS GPS location information sent via GPRS with SMS backup for transmission of alarm messages, in poor communication conditions. Two-way audio: microphone is sensitive up to 2 m LED status indication: low battery, GPS, GSM coverage and connection	Wireless communication Connect to the internet Always online and connect to the database Speech communication Detect a change in angle, orientation and impact to differentiate a fall from normal ADLs	Detect a fall and user lying unconsciously Fit with emergency button When standing on a table, can be used as a “knock-over” alarm to summon assistance Detect wandering using radio signal strength Detect potential hypothermia (low temperature for prolonged time)	Automatic fall detection Fit with emergency button Full waterproofInclude neck cord with built-in safety release mechanism and belt-clip Superior transmission range Smart anti-collision algorithm Support multiple simultaneous transmissionsPower Code ID factory-selected from 16 million possible code combinations Visible and transmitted low battery indication Available in several optional frequencies in compliance with international standards	Automatic fall detection Emergency call Patient call Waterproof An in-built cancellation button to cancel the call if necessary Can be set to vibrate	Automatic fall detection Waterproof Low battery detection Compatible with climax medical alarm panels Pendant style Operating temperature: −10 to 40 °C Humidity: up to 90% non-condensing	Adjustable sensitivity with three different levels to suit individual needs and circumstances Ergonomic alert button enabling people with visual impairments or limited dexterity to raise a call for help Crescendo audible alert and status LED provide user with reassurance the device has registered a fall Not-worn alert which will notify the monitoring center if the fall detector has failed to move, indicating that it may not have been worn Cancellation button enabling the wearer to cancel activations if required, reducing the number of false calls (this function can be disabled if required) Water resistant to IP67 standard enabling the fall detector to be worn in the bathroom Automatic low battery warnings Long-life, replaceable battery in easy-open compartment to enable simple replacement	Automatic fall detection through multiple accelerometers and sensors Programmable transmission delay time of 0–9 s Can cancel help calls or false alarms with an 8-second press of the help button Low battery detection and supervision Able to answer incoming phone calls on the home phone line Waterproof design Can be worn around the neck as a pendant Adjustable lanyard with snap closure Operating temperature: −10 to 40 °C Humidity: up to 90% non-condensing	Intelligent personalized alerting Unique user identification Rule engine personalized by individual Alert & trend on any combination of factors Health monitoring, trending and alerting Ease of installation and support Software as a service Network independent Built on open standards and AP IsInteroperability Preserve investment in existing systems while extending life with new capabilities Location awareness and support Personalized wander management solutions built into the system with ability to integrate to door locks	Automatic fall detection and call for help Waterproof Easily accessible Neck cords are designed to break away in the event of an emergency and are easily replaced if damaged or soiled Can call for help within the range of the communicator	Triggered by change of angle and impact of a fall Wear on front or side waist Green light and two “beeps” indicate that the fall detector has been activated Fit with emergency button	5 user-selectable levels of sensitivity 3 event logs; impacts, pre-alarms & alarms Weekly rental Detect fall, fall recovery, stumble, and trip Fit with emergency button	Automatically call for help when falling Fit with emergency button	LCD display Re-chargeable battery Automatic fall detection Fit with emergency button	5 sensitivity settings Daily battery self-test & low battery reporting	Automatic fall detection Heart rate monitoring Activity monitoring Fall prevention Location alarms Call button Rechargeable battery	Detect fall when the detector is in a position of 70° or greater from vertical
